# Bee Pollen: Chemical Composition and Therapeutic Application

**DOI:** 10.1155/2015/297425

**Published:** 2015-03-11

**Authors:** Katarzyna Komosinska-Vassev, Pawel Olczyk, Justyna Kaźmierczak, Lukasz Mencner, Krystyna Olczyk

**Affiliations:** ^1^Department of Clinical Chemistry and Laboratory Diagnostics, School of Pharmacy and Division of Laboratory Medicine in Sosnowiec, Medical University of Silesia, Jednosci 8, 41-200 Sosnowiec, Poland; ^2^Department of Community Pharmacy, School of Pharmacy and Division of Laboratory Medicine in Sosnowiec, Medical University of Silesia, Kasztanowa 3, 41-200 Sosnowiec, Poland

## Abstract

Bee pollen is a valuable apitherapeutic product greatly appreciated by the natural medicine because of its potential medical and nutritional applications. It demonstrates a series of actions such as antifungal, antimicrobial, antiviral, anti-inflammatory, hepatoprotective, anticancer immunostimulating, and local analgesic. Its radical scavenging potential has also been reported. Beneficial properties of bee pollen and the validity for their therapeutic use in various pathological condition have been discussed in this study and with the currently known mechanisms, by which bee pollen modulates burn wound healing process.

## 1. Introduction 

Apitherapeutics are natural agents which contain groups of chemical compounds with approved action and range of activity. The chemical composition of one of the most known apitherapeutics, bee pollen, depends strongly on the plant source and geographic origin, together with other factors such as climatic conditions, soil type, and bees race and activities [1, 2]. In the composition of the bee pollen, there are about 250 substances including amino acids, lipids (triglycerides, phospholipids), vitamins, macro- and micronutrients, and flavonoids [1, 2].

Bee pollen is used in the apitherapeutic treatment as it demonstrates a series of actions such as antifungal, antimicrobial, antiviral, anti-inflammatory, immunostimulating, and local analgesic and also facilitates the granulation process of the burn wound healing [3, 4].

Bee pollen is a raw material from which bees produce bee bread. They collect pollen from plant anthers, mix it with a small dose of the secretion from salivary glands or nectar, and place it in specific baskets (corbiculae) which are situated on the tibia of their hind legs. These are called pollen loads. The field bees collect and transport the bee pollen to the hive [5, 6].

In the hive, the collected pollen, dampened with saliva and fragmented by flightless bees, is packed in honeycomb cells. Next, the surface of the collected pollen is covered with a thin layer of honey and wax. The substance which has been created is bee bread which undergoes anaerobic fermentation and is preserved thanks to the arising lactic acid. Bee bread constitutes the basic protein source for the bee colony. Moreover, it is also the source of nutritional and mineral substances for royal jelly produced by worker bees [5, 6].

The bee pollen occurs in the anthers of seed plants in the form of 2,5–250 *μ*m grains. The grain dust is encompassed by a double-layered cell wall. The internal cell wall is called intine, while the external one is called exine. The exine defines itself by a strong resistance to physicochemical factors. Furthermore, on its surface, there are numerous pores and furrows as well as a layer of balsam which all facilitate sticking of pollen to bees' abdomens [5].

Pollen grains, depending of the plant species, differ in shape, color, size, and weight. The grain shapes are diverse: round, cylindrical, bell-shaped, triangular, or thorny [7]. Their weight is equal to a dozen or several dozens of micrograms. The majority of pollens consist of single grains which are sometimes joined with two or more grains [7].

The color of the pollen is varied ranging from bright yellow to black. The pollen basket, which is brought to the hive, usually consists of the pollen from one plant. However, it sometimes happens that the bees collect pollen from many different plant species. The group of plants from which only pollen is collected includes poppy, corn, and lupine, while from other melliferous plants bees collect both nectar and pollen. Bees do not collect pollen from grass. However, they may occasionally collect fungal spores from moldy plants [8, 9].

## 2. Chemical Composition of Pollen

Pollen is quite a varied plant product rich in biologically active substances. 200 substances were found in the pollen grains from different plant species. In the group of basic chemical substances, there are proteins, amino acids, carbohydrates, lipids and fatty acids, phenolic compounds, enzymes, and coenzymes as well as vitamins and bioelements [10, 11].

Pollen contains 22,7% of protein on average, including 10,4% of essential amino acids such as methionine, lysine, threonine, histidine, leucine, isoleucine, valine, phenylalanine, and tryptophan. These protein elements are life essential and the organism cannot synthesize them by itself. Moreover, in the pollen, there are significant amounts of nucleic acids, especially ribonucleic one. Digestible carbohydrates occur in the pollen in the amount of 30,8% on average. Reducing sugars, mainly fructose and glucose, are present in this product in about 25,7% [12–15].

Among lipids, which are present in the pollen in the amount of about 5,1%, the ones which should be mentioned in the first place are essential fatty acids (EFAs). Acids such as linoleic, *γ*-linoleic and archaic exist in the amount of 0,4%. Phospholipids amount to 1,5%, while phytosterols, especially P-sitosterol, are present in the amount of 1,1% [16].

Another group constituted phenolic compounds which amount to 1,6% on average. This group includes flavonoids, leukotrienes, catechins, and phenolic acids. Among flavonoids occurring in the pollen in 1,4%, there are mainly kaempferol, quercetin, and isorhamnetin, while in the group of phenolic acids, 0,2%, there is mainly chlorogenic acid [17].

Pollen is characterized by a quite significant content of triterpene bonds. The most frequent compounds are oleanolic acids, 3-ursolic acid, and betulin alcohol [12, 13].

Moreover, vitamins and bioelements also belong to valuable substances. Pollen is quite a significant source of vitamin both fat-soluble 0,1%, such as provitamin A and vitamins E and D, and water-soluble 0,6%, such as B1, B2, B6, and C, and acids: pantothenic, nicotinic and folic, biotin, rutin, and inositol. Their total amount is equal to 0,7% in the whole product.

Bioelements are present in about 1,6%, including macronutrients (calcium, phosphorus, magnesium, sodium, and potassium) and micronutrients (iron, copper, zinc, manganese, silicon, and selenium). The latter one exists in the amount of 0,02% [10–13].

According to the latest National Data, the average content of main ingredients in the air-dried pollen (at the temperature 40°C) amounts to such values as follows: proteins, 32,8%, including essential amino acids, 11,5%, and reducing sugars, 40,7%, including sucrose, 3,7%, lipids, 12,8%, vitamin C, 0,19%, *β*-carotene, 0,07%, and bioelements, 4,0%.

Special devices, pollen traps, are used to collect pollen baskets. The general rule of their functioning is to take the part of the pollen basket from field bees returning to the hive. Therefore, there are different dividers on the returning route of bees. The bees must force their way through and, consequently, lose the part of the pollen basket which falls into special containers. There are different types of pollen traps: outlet, bottom-board, slice, and top-frame ones in which perforated screens or grids with appropriately small holes are used. Their size is about 5 mm.

The loss of pollen mobilizes the bees. It increases both the number of field bees and the number of flights. The amount of pollen collected from one colony during one day amounts to 50–250 g. According to National Data, one bee colony gives 1 to 7 kg of pollen a year [12, 13].

Bee bread is collected by scratching it from the combs with a special fork and then attenuated with warm honey in the ratio of 1 : 5. After leaving the mixture for several days, bee bread falls down on the bottom of the container as it is heavy and separates itself from honey. After putting it into jars, the product is closed tightly and stored in a cool and dark place [18].

## 3. Activity and Biological Properties of Pollen

Experimental pharmacological studies, conducted on rats and rabbits, showed that pollen has an hypolipidemic activity decreasing the content of plasma total lipids and triacylglycerols. Additionally, the decrease of lipid concentration in the serum correlated with the content of such hormones as insulin, testosterone, and thyroxine, which are responsible for a higher lipid metabolism [19, 20].

Clinical studies confirmed the hypolipidemic activity of pollen. It made the content of the above-mentioned lipid substances decrease in the blood serum in patients from 20 to 35% [21]. It was also successfully applied in hyperlipidemia and atherosclerosis. In patients who did not react on antiathersclerotic drug, Grofibrat (fenofibratum), pollen lowered the level of lipids and cholesterol from 20 to 30% and decreased the clumping of blood platelets for 30% [22, 23]. In patients suffering from arteriosclerosis with a significant myopia and partial optic atrophy, pollen lowered the level of cholesterol in blood serum and increased the field of view and stabilized the visual acuity [24].

Pollen and its extracts, fat-soluble ones in particular, are successfully applied in postinfarction conditions as well as in systemic circulation disorders and arterial hypertension. Moreover, small doses of pollen given to older people allow both the inhibition of the atherosclerotic changes of blood vessels and improvement of cerebral blood flow [25].

The hypoglycemic activity of pollen is mainly ascribed to the presence of unsaturated fatty acids, phospholipids, and phytosterols. Furthermore, a decreased ability of platelet aggregation and increased fibrinolytic system activity was confirmed in people who take pollen. It indicates the antiatherosclerotic effect which protects from heart diseases and brain strokes [26].

The wide-ranging and well-documented studies on animals also unambiguously showed detoxifying action of pollen. The rats were poisoned with organic solvents such as carbon tetrachloride and trichlorethylene, as well as ethionine and ammonium fluoride, both causing a deep damage of liver cells, and galactosamine, which imitate the changes of viral hepatitis, ethanol, and allyl alcohol, which induce steatosis and cirrhosis, and with drugs: paracetamol and hydrocortisone. Under their influence, very high levels of enzymes such as alanine and aspartate transaminase, acid phosphatase, and bilirubin were assayed [27]. Pollen lowered the level of these substances in the blood serum even to physiological values, which proves the therapeutic properties of this product in reference to liver tissue. However, when it was administered with toxic substances, it protected liver cells from their harmful effect, which indicates, in turn, its ability to prevent toxication. In the detoxifying process, an important role is played by polyphenols, mainly flavonoids and phenolic acids [28–31].

The detoxifying activity of pollen and bee bread in phenomena such as occupational diseases, heavy metal contamination, industrial gases and dusts, and drugs (e.g., antirheumatic and anti-inflammatory preparations and antibiotics) should also be mentioned [32].

Pollen is also characterized by a high anti-inflammatory activity. Its magnitude is compared to such nonsteroidal anti-inflammatory drugs as naproxen, analgin, phenylbutazone, or indomethacin [33].

The mechanism of anti-inflammatory effect is about inhibiting the activity of cyclooxygenase and lipoxygenase, the enzymes responsible for turning arachidonic acid into such toxic compounds as prostaglandin and leukotrienes, inducing acute and chronic inflammatory conditions in tissues. The experimental research shows that a concentrated extract of pollen, in the dose of 50 mg for rat's body weight, eliminates in 75% the swelling of the given animal's paw induced by carrageenan administration. The elements responsible for such activity are flavonoids and phenolic acids as well as fatty acids and phytosterols [34]. Pollen is recommended in acute and chronic inflammatory conditions, initial degenerative conditions, and cholestatic liver diseases as well as in toxic and posttraumatic damages of this organ [33, 34].

Bee pollen has also been proposed as a valuable dietary supplement. Animal feeding experiments with pollen have also been carried out. It was proven that mice and rats, fed with pollen, showed a higher vitamin C and magnesium content in thymus, heart muscle, and skeletal muscles as well as a higher hemoglobin content and greater number of red blood cells when compared to animals given standard feed. Moreover, pollen also lengthened the life span of experimental animals [35–37].

In starved animals and those being on a nonvitamin diet, pollen caused faster weight gains than a normal diet. The research proves that pollen has a high nutritional value as well as a property of fast supplementing the nutritional deficiencies in animals' organisms. The components playing the vital role in the process are dispensable amino acids, vitamins, and bioelements [36, 38].

Nutritional properties of pollen and the regulating metabolic processes are used, among others, in the cases of children's lack of appetite, developmental delay, and malnutrition of children and adults. Moreover, it is recommended to administer pollen in the recovery period, after surgeries, and to people working hard physically and mentally [36, 38].

Furthermore, the adaptogenic properties of pollen, which are based on increasing the resistance to harmful physical, chemical, and biological factors, were also indicated: it is both (1) increasing the physical fitness of the organism in excessive physical burden, affecting the central nervous system by improving brain functions, such as memory, learning, comprehending, thinking, and ability to concentration, and (2) increasing the immune system strengths against infection en route boosting the immunological system [39].

It has been also shown that pollen ethanol extracts have quite a strong antibiotic activity that is still being effective on the pathogen for human Gram-positive bacteria, for example,* Staphylococcus aureus*, and Gram-negative bacteria, including* Escherichia coli, Klebsiella pneumoniae, Pseudomonas aeurgionsa,* and on fungi such as* Candida albicans*. The responsibility for this activity lies in flavonoids and phenolic acids [40, 41].

Recent research indicates that pollen has an antiallergic activity. It protects mast cells of the organisms from degranulation that is from releasing histamine which is the exponent of allergic reactions. For instance, releasing histamine from mast cells, induced by the serum containing anti-IgE antibodies, was inhibited by pollen in 62% [42].

The literary data point out that pollen seals capillaries, removes swellings of cardiovascular and renal origin, and has a spasmolytic effect on smooth muscles especially in the range of bladder and urethra [43].

The beneficial effect of pollen in inflammatory conditions of prostate gland is known for a long time. Clinicians confirm that, in nonbacterial prostate inflammations, pollen improves the condition of patients effectively removing the pain. The positive effect was found in benign prostatic hyperplasia cases. In the initial stage of the prostate cancer, the improvement was also found. However, when pollen was administered alongside chemotherapeutic agents, the number of people who felt a significant therapeutic effect significantly increased [44–47].

Pollen, administered alongside antidepressants, enables the lowering of their doses and improves the overall condition in a short period of time. Due to this fact, there are fewer cases of drug addictions or occurrences of side effects. Owing to its nutritional and tonic properties as well as improvement of blood supply to nervous tissue, pollen boosts mental capacity and strengthens the nervous system weakened by stress or overworking [48–50]. Therefore, pollen is effective in treating physical and mental overtiredness, asthenia, and apathy.

Particularly good effects are gained in depressions caused by decreased life energy, especially in older people. Long-term use of pollen, even in small doses, enables gradual mood improvement, restores the desire to live, and strengthens the organism physically [50].

Good results of treatment with pollen and bee bread were gained in geriatrics in the symptoms of early old age as well as in neurasthenic inertia in older people. Pollen is a vital element in treating chronic alcoholic disease. Small doses of pollen and tranquilizers together with fluid administration enable both the alleviation of the abstinence symptoms and significant reduction of their duration. Deficiencies of many substances such as proteins, vitamins, and bioelements, magnesium in particular, which occur in chronic alcoholism, are supplemented to a great extent by pollen [17, 51].

## 4. Routes of Administration and Dosing

In adults, 20–40 g is applied therapeutically every day. If a teaspoon is 7,5 g of pollen, it can be concluded that one dose is 3–5 teaspoons of this product for adults and 1-2 teaspoons for children. Pollen is usually taken 3 times a day before eating. The time of treatment is 1–3 months, but it can be repeated 2–4 times a year. The most appropriate period for treatment is between winter and spring and between summer and autumn. Generally, a smaller dose of pollen is used in the combination therapy, alongside other medicaments and in chronic diseases [52].

Bee bread, as a product of a stronger action than pollen, is usually administered in smaller amounts or for a short period of time. Romanian researchers, in the therapy of a chronic hepatitis, gained the same results for bee bread used in the amount of 30 g daily during a month and for pollen in the exactly the same dose administered for 3 months.

In order to increase the digestibility of the organism, pollen grains are shredded by grinding or are subjected to warm water. In the water environment, pollen grains become swollen and, after 2-3 hours, crack and, consequently, release their values. Milk, fruit, and vegetable juices are also used for this purpose. (Ground) pollen may be mixed with many products in the ratio from 1 : 1 to 1 : 4 with the use of honey, butter, cottage cheese, yoghurt, jams, glucose, and others. Mixed pollen is taken in the amount of 1 teaspoon 3 times a day. In many diseases, however, enzymatic pollen is recommended for use.

To sum up, it should be emphasized that unshredded pollen, accurately chewed before swallowing, is used by the organism only in about 10–15%. After mechanical shredding or natural release, the accessibility of biological pollen increases to 60–80% [52, 53].

## 5. Pollen in Burn Wounds Treatment

Apitherapy is becoming more and more recognized among contemporary and conventional treatment methods as it uses therapeutic effect of standardized, pharmacologically active fractions obtained from bee products. Literary data indicate that the antioxidating, immunomodulating, epithelialization accelerating properties and bacteriostatic and anesthetic characteristics and the advisability of its application in burn wound treatment are confirmed [54, 55]. Furthermore, the equally important fact is that pollen has a strong anti-inflammatory effect, shortens the healing time, decreases the discomfort of both the duration period and the intensity of ailments, and is definitely less costly. The mechanism of the inflammatory effect is about inhibiting the activity of enzymes which are responsible for the development of inflammatory process mediators in tissues. Flavonoids and phenolic acids are mainly responsible for such actions, but fatty acids and phytosterols also take part in this process [33, 34].

What is more, kaempferol, which is included in pollen, thanks to its ability to inhibit the activity of two enzymes: hyaluronidase, which is the enzyme catalyzing depolymerisation of hyaluronic acid, and elastase, which hydrolyses elastin, strengthens the connective tissue and seals blood vessels. This results in decreased transudates, inflammatory reactions, and swellings. Blood circulations in the vessels improve and, therefore, skin becomes moistened and tight. The antiedematous, anti-inflammatory, and analgesic action of flavonoids may also result from a different compound bioactivity; for example, quercetin, by inhibiting the activity of histidine decarboxylase, lowers the histamine level in the organism. Moreover, inhibiting the cascade of arachidonic acid metabolism, which in turn lowers the level of proinflammatory prostaglandins and gives the anti-inflammatory effect, removes local pain and prevents platelet aggregation [52, 56–58].

It should also be mentioned that one of the factors interrupting the healing process of wounds is infection. Particularly susceptible to infections are postburn wounds, which were the subject of previous studies on experimental therapy of burns with propolis. Extensive burns are the gates of infection for many microorganisms, while the necrotic tissues are a very good environment for such microorganisms to develop [54]. The therapeutic mechanism of apitherapeutics is based, among others, on antimicrobial activity and on inducing processes of regeneration of damaged tissues. These properties indicate the possibility of using apitherapeutics in burn wound treatment and ulcerations of different etiology [40, 59]. The conducted studies, that have not been published yet, prove that the ointment with bee pollen extract has an antimicrobial activity regarding the bacterial flora of postburn wounds. Moreover, the apitherapeutic method of burn wound treatment, including topical application of the bee pollen ointment, is additionally deprived of undesirable effects and is alternative to topical burn wound treatment.
